# The Iron–Sulfur Cluster of Bacterioferritin‐Associated Ferredoxin (Bfd): a “Biological Fuse” that Prevents Oxidative Damage to Cells?

**DOI:** 10.1002/anie.202511340

**Published:** 2025-06-25

**Authors:** Justin M. Bradley, Aiden M. Carter, Zinnia Bugg, Simon C. Andrews, Nick E. Le Brun

**Affiliations:** ^1^ Centre for Molecular and Structural Biochemistry School of Chemistry University of East Anglia Norwich Research Park Norwich NR4 7TJ UK; ^2^ School of Biological Sciences University of Reading, Whiteknights Reading RG6 6EX UK

**Keywords:** Bfd, Electron transfer, Ferritin, Iron homeostasis, ROS

## Abstract

Iron is in an essential micronutrient in living systems. However, it is also potentially toxic and so the concentration of chelatable iron within cells is tightly regulated to prevent catalytic formation of harmful reactive oxygen species (ROS). Ferritins play a key role in iron homeostasis by storing excess iron as an insoluble ferric mineral within the protein. When bacterial cells become iron deficient, this store may be accessed by reduction/solubilisation of the iron. Bacterioferritins utilise heme, bound at an inter‐subunit site, to support electron transfer to the stored mineral. Electrons for heme reduction are shuttled from NADPH via Bfd, a [2Fe–2S] cluster‐containing ferredoxin. This raises the paradox that the synthesis of an iron‐dependent protein co‐factor is required under conditions of iron‐deficiency so that stored iron can be utilised. Here, we show that exposure of Bfd to ROS suppresses the capacity of the protein to stimulate iron release from bacterioferritin. We propose that reliance of iron release on Bfd evolved to ensure that chelatable iron levels do not increase under oxidative stress conditions. Thus, the Bfd iron–sulfur cluster functions as a “biological fuse” in providing a fail‐safe that immediately halts iron release once ROS accumulate to damaging concentrations.

## Graphical abstract

When cellular iron is low, bacterioferritin (Bfr) releases its stored iron through a reductive process dependent on the [2Fe–2S] ferredoxin Bfd. Why accessing stored iron should depend on an iron‐requiring protein when iron is scarce is unclear. Here, we show that the Bfd [2Fe–2S] cluster is sensitive to oxidative stress, suggesting that it may act as a “biological fuse”, shutting down iron release under conditions where free iron is toxic.

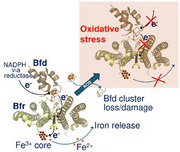

**Abbreviations**
Bfr: bacterioferritin
Bfd: bacterioferritin‐associated ferredoxin
CD: circular dichroism
DTT: dithiothreitol
Fe–S: iron‐sulfur
FoC: ferroxidase centre
GSH: glutathione
MES: 2‐(N‐morpholino) ethanesulfonic acid
MOPS: (3‐(N‐morpholino) propanesulfonic acid
RNS: reactive nitrogen species
ROS: reactive oxygen species
SOD: superoxide dismutase
Tris: tris(hydroxymethyl)aminoethane

## Introduction

Many proteins that carry out essential biological processes rely upon iron‐containing co‐factors. The simplest of these co‐factors, termed non‐heme iron, comprises the binding of the metal to carboxylate‐ or histidine‐rich sites, and are typically involved in the binding or activation of O_2_.^[^ ^1^, ^2^ ^]^ Heme consists of iron bound at the centre of a porphyrin macrocycle and is a versatile co‐factor utilised in a variety of electron transport and catalytic roles.^[^ ^3^ ^]^ Iron–sulfur (Fe–S) clusters, a combination of iron and sulfide bound at cysteine‐rich motifs, are thought to be the most ancient of the iron‐containing co‐factors.^[^ ^4^ ^]^ They display a versatility of function similar to that of heme, and early life evolved to become reliant on their chemistry prior to the oxygenation of the atmosphere.^[^ ^5^ ^]^ Whilst reliance upon Fe–S clusters to support life persists in aerobic environments, it is acknowledged that both sulfide and cysteine participate in reactions with O_2_ not mirrored in the chemistry of porphyrin or non‐heme iron centres.^[^ ^4^ ^]^ As such, Fe–S clusters can be considered to be generally more susceptible to damage by O_2_ and reactive oxygen species (ROS) than other common iron‐containing co‐factors.

Oxygenation of the atmosphere led to difficulties in the utilisation of iron in biological processes beyond damage to Fe–S clusters. In anaerobic and acidic environments iron is readily available because the Fe^2+^ oxidation state, the thermodynamically stable form in these conditions, is soluble. However, at pH > 3, aqueous Fe^2+^ is readily oxidised to Fe^3+^ in the presence of O_2_,^[^ ^6^ ^]^ leading to its deposition in insoluble minerals such as hematite. Therefore, despite being the fourth most abundant element by mass in the Earth's crust, bioavailability of iron is sufficiently poor that it is very often a growth‐limiting micronutrient. Furthermore, superoxide and peroxide are inevitable by‐products of aerobic respiration and iron can act as a catalyst for their conversion into O_2_, hydroxide and the highly toxic hydroxyl radical (OH^•^) by cycling between its + 2 and + 3 oxidation states,^[^ ^7^ ^]^ leading to irreversible damage to proteins, protein co‐factors, nucleic acids, and lipids. Superoxide and peroxide can both promote the disintegration of Fe–S clusters with the iron liberated in this process potentially exacerbating the oxidative burden on the cell via its interaction with ROS.^[^ ^8^ ^]^ In addition to direct damage to biological molecules, the combination of ROS with nitric oxide generates reactive nitrogen species (RNS) such as nitrogen dioxide and peroxynitrite, themselves capable of degrading Fe–S clusters, damaging nucleic acids and causing loss of protein function due to covalent modification of amino acid sidechains, thereby destabilising secondary structure.^[^ ^9^ ^]^ As a consequence of this potential toxicity, the concentration of chelatable iron within cells is tightly regulated to minimise the risk of oxidative damage. However, any excess above that required to maintain the iron‐containing proteome is typically not excreted from cells.^[^ ^10^ ^]^ In most organisms excess iron is sequestered within the hollow protein shell of members of the ferritin family for use when cytosolic iron levels become depleted.^[^ ^11^ ^]^

The cage‐forming ferritins are multimeric proteins^[^ ^12^ ^]^ that spontaneously self‐assemble under physiological conditions. Hydrophilic channels that penetrate the protein coat sequester excess cytosolic Fe^2+^ from solution,^[^ ^13^, ^14^, ^15^, ^16^ ^]^ and the iron is subsequently guided to diiron catalytic sites, called ferroxidase centres (FoCs).^[^ ^17^, ^18^, ^19^ ^]^ Oxidation of Fe^2+^ at the FoCs is coupled to the reduction of either O_2_ or peroxide,^[^ ^20^ ^]^ resulting in the synthesis of Fe^3+^‐oxo species. Ferric iron is then released to nucleation sites on the inner surface of the cage,^[^ ^13^, ^14^, ^21^, ^22^, ^23^ ^]^ forming the precursor to the insoluble ferrihydrite‐like mineral core. Ferritin iron stores are accessed when the availability of environmental iron decreases and the cytosolic iron level is below that required to maintain the metal‐binding status of the iron‐containing proteome. This occurs either by degradation of the protein or by reduction of the Fe^3+^ within the encapsulated mineral back to the soluble Fe^2+^ state.^[^ ^24^, ^25^, ^26^, ^27^, ^28^, ^29^, ^30^, ^31^ ^]^ The details of these iron‐release processes are generally poorly understood, the notable exception being the bacterioferritins (Bfrs).^[^ ^32^, ^33^ ^]^

**Figure 1:**
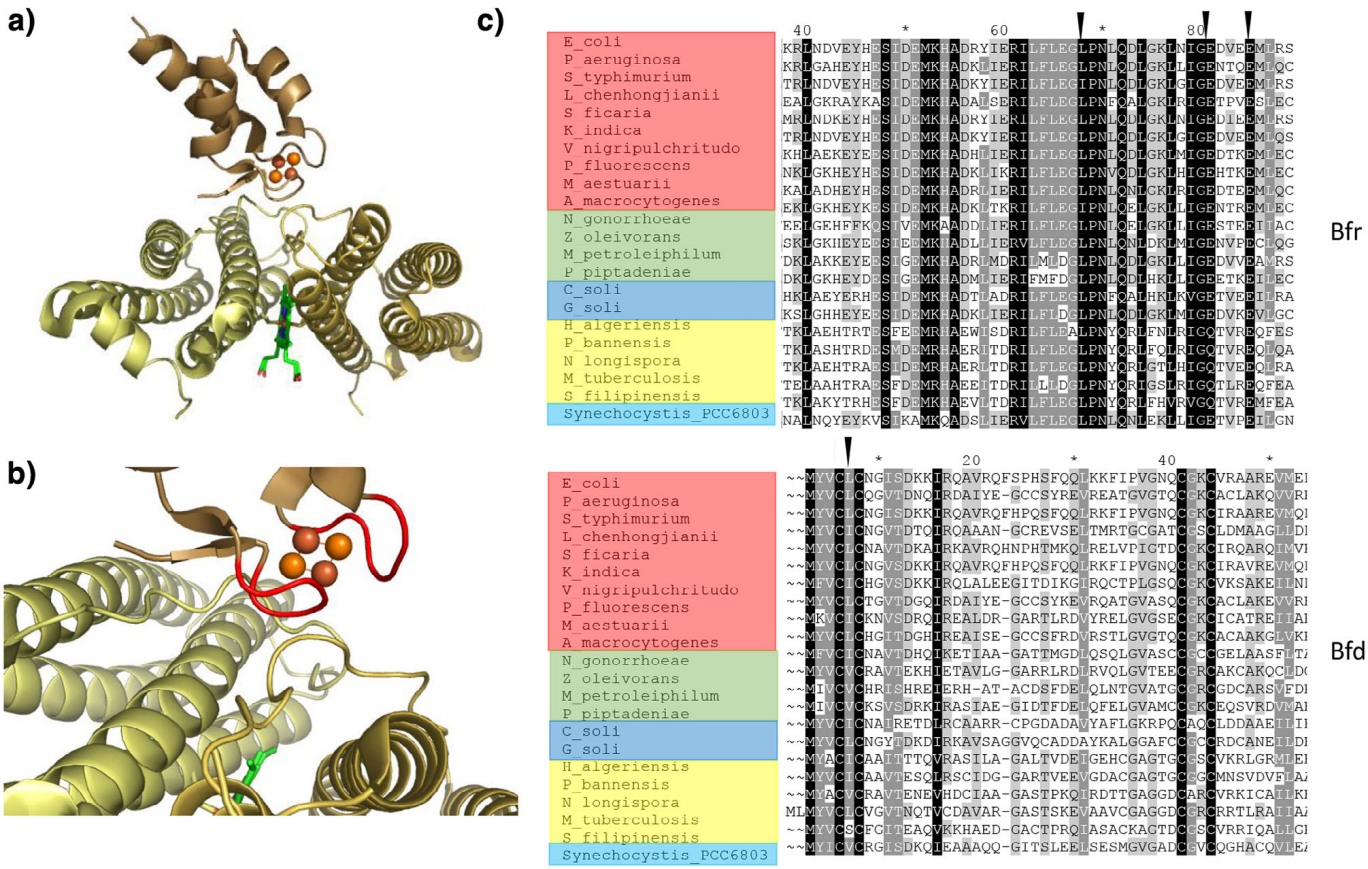
Conservation of the Bfr–Bfd interface. a) Binding of *P. aeruginosa* Bfd to the outer surface of the cognate Bfr, viewed along the long axis of the Bfr monomers. b) Rotated view of *P. aeruginosa* Bfd bound to Bfr with the loops L1 and L3 of Bfd that stabilise the complex with Bfr highlighted in red c) Partial sequence alignments of randomly selected putative Bfr and Bfd proteins from γ‐ (red), β‐ (green) and α‐ (blue) proteobacteria, actinomycetes (yellow) and cyanobacteria (cyan) with the positions of residues identified as hotspots stabilising the Bfr:Bfd complex^[^ ^38^ ^]^ marked with black triangles. All three hotspot residues on Bfr are strictly conserved whilst Leu5 of P. aeruginosa is conserved or conservatively substituted by Ile or Val. Panels (a) and (b) were generated from PDB entry 4E6K.^[^ ^32^ ^]^

Each face of the rhombic dodecahedral cage of ferritins is composed of a subunit dimer. Bfrs are unique among ferritins in that they are heme proteins, with the heme sites located between the two monomers of each face, towards the inner surface side of the protein coat^[^ ^34^ ^]^ (Figure 1). Iron stored within Bfr is released via reduction of the mineral core, with the heme acting as a conduit for electrons to access the mineral both directly and via the FoC sites.^[^ ^33^, ^35^ ^]^ Electrons for the reduction of Bfr iron stores are thought to ultimately be derived from the NADPH/NADP^+^ couple, and are passed to the heme of Bfr via a ferredoxin reductase and a [2Fe–2S] cluster‐containing ferredoxin, bacterioferritin‐associated ferredoxin (Bfd).^[^ ^32^ ^]^

The consequences of disrupting the Bfd:Bfr interaction in *Pseudomonas aeruginosa* demonstrated that the requirement to pass electrons to the hemes of Bfr via the Bfd [2Fe–2S] cluster cannot be circumvented in vivo.^[^ ^36^, ^37^ ^]^ This raises an apparent paradox: under conditions of iron deficiency, bacterial cells are required to commit scarcely available iron to the synthesis of an iron‐containing co‐factor in order to access their Bfr‐encapsulated store of this essential micronutrient. However, in vitro assays demonstrated that flavins such as FMN are also capable of driving the reductive release of Bfr encapsulated iron in a heme‐dependent manner.^[^ ^33^, ^35^ ^]^ Thus, the reliance of iron release from Bfr on the Bfd cluster in vivo is not an absolute physical requirement. Nevertheless, the widespread conservation of the Bfr:Bfd interface across many bacteria (Figure 1c) suggests that this requirement confers some advantage in terms of survival.

We hypothesise that the inherent vulnerability of Fe–S clusters to ROS‐induced damage results in the termination of Bfd‐driven iron release under conditions where ROS accumulate, thereby preventing the exacerbation of oxidative stress due to increased levels of available Fe^2+^. Here, we employed a combination of absorbance and circular dichroism spectroscopies, together with kinetic measurements of in vitro iron release from Bfr, to demonstrate that exposure of Bfd to ROS leads to degradation of the ferredoxin cluster, and consequent abolition of iron‐release activity. A similar vulnerability was not observed following equivalent treatment of the iron‐free co‐factor FMN.

## Results and Discussion

### The [2Fe–2S] Cluster of Bfd is Damaged by Exposure to ROS

Stability of the Bfd Fe–S cluster was determined using the xanthine oxidase system to generate ROS in situ,^[^ ^39^ ^]^ whilst monitoring the absorbance and circular dichroism (CD) of the ferredoxin which was isolated with its [2Fe–2S] cluster in the oxidised state. Removal of the Bfd cluster resulted in abolition of absorbance and CD at wavelengths greater than 300 nm. In contrast, reduction of the cluster led to bleaching of sharp features in the absorbance spectrum at 425 and 475 nm but broad absorbance features in the 400 to 600 nm range remained. In the CD spectrum, reduction led to loss of the negative feature at 550 nm, together with a change in sign of the positive feature at 435 nm and the appearance of a sharp negative band at 330 nm^[^ ^22^ ^]^ (Figure S1). Degradation and reduction of the oxidised cluster are therefore readily distinguished utilising a combination of absorbance and CD spectroscopy.

The concentration of superoxide and peroxide generated during the hydroxylation of 1 mM hypoxanthine by xanthine oxidase were first quantified by monitoring the reduction of ferric cytochrome *c* and through the Amplex Red assay, respectively, with 40 µM superoxide and 57 µM peroxide detected (Figure S2). Therefore, employing a solution 20 µM in Bfd clusters during stability assays ensured that ROS generated by xanthine oxidase activity was in excess of Fe–S cluster concentration, and superoxide generated in equivalent stoichiometry to cluster bound iron, with peroxide in slight excess. Control experiments in which 20 µM Bfd was exposed to 210 µM O_2_, or 1 mm of both xanthine and uric acid, for an hour confirmed the stability of the Bfd cluster to the aerobic conditions required for xanthine oxidase activity and to the products of hypoxanthine hydroxylation (Figure S3).

**Figure 2:**
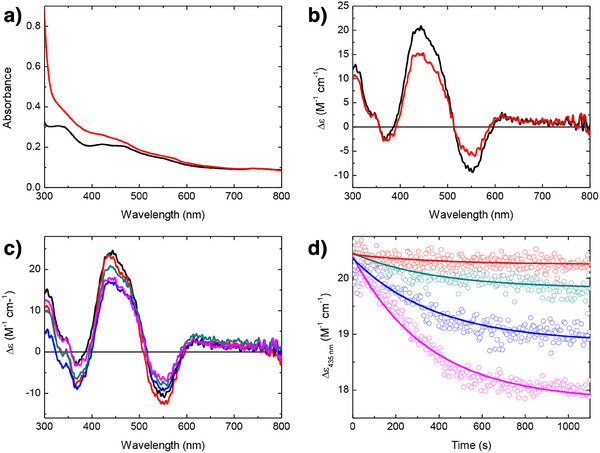
ROS‐induced decay of the Bfd [2Fe–2S] cluster. a) The absorbance spectra of Bfd as‐isolated (black) and following exposure to xanthine oxidase, hypoxanthine and 210 µM O_2_ for 30 min (red). b) The CD spectra of the samples from (a). c) The CD spectra of Bfd following exposure to xanthine oxidase and hypoxanthine for 30 min in anaerobic solution (black), or solution containing 60 (red), 125 (green), 170 (blue) or 230 (magenta) µM O_2_. d) The time dependence of the 435 nm CD response of the Bfd cluster following the addition of hypoxanthine to the samples from (c) together with exponential functions describing the decay.

In contrast to the above, addition of hypoxanthine to 20 µM Bfd in the presence of xanthine oxidase and O_2_ led to significant changes in both the absorbance and CD spectra of Bfd. Interpretation of the absorbance spectra were complicated by interfering contributions from the large excess of hypoxanthine, although the hypoxanthine did not impact the CD spectra (Figure 2). Decreases in CD intensity at 550 and 435 nm were not accompanied by the appearance of a negative band at 330 nm, indicative of degradation rather than reduction of the cluster. The time dependence of the CD signal of the cluster indicated that breakdown occurred over a period of 10 min. However, the spectra acquired at the endpoint of the reaction revealed that cluster breakdown was incomplete. Given that hypoxanthine was present in large excess, this suggests that reactivity was limited by O_2_ availability to the xanthine oxidase system. This was investigated by repeating the cluster stability assay at a range of dissolved O_2_ concentrations, achieved by mixing aerobic (250 µM dissolved O_2_) and anaerobic buffer in various ratios (Figure 2).

The extent of cluster degradation increased with dissolved O_2_ concentration, confirming that O_2_ was limiting for this process, such that approximately 30% of CD intensity associated with the cluster was lost following incubation with hypoxanthine and xanthine oxidase at the maximum achievable O_2_ concentration (230 µM). In contrast, the dissolved O_2_ concentration had no significant effect on the rate of cluster degradation, with each trace well described by a first order process with a rate constant of 0.16 min^−1^, indicating that the turnover of xanthine oxidase was not rate‐limiting for this process under the conditions utilised.

### Glutathione and Fe^2+^ Exacerbate ROS−Induced Damage to Bfd Clusters

**Figure 3:**
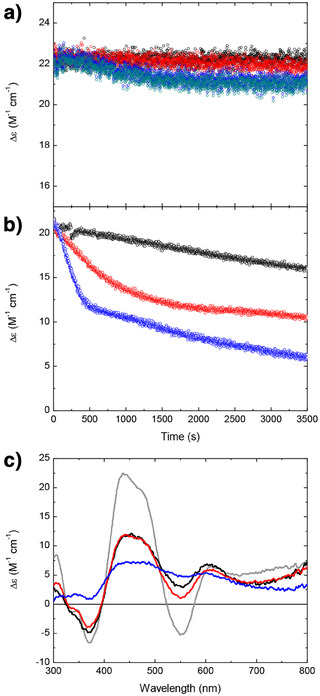
The effect of Fe^2+^ and glutathione on ROS‐induced decay of the Bfd cluster. a) Time‐dependence of the 425 nm CD response of the Bfd [2Fe–2S]‐cluster anaerobically in the presence of 10 µM Fe^2+^ (black) or 2 mm glutathione (red). Equivalent samples in the presence of 210 µM O_2_ are represented by blue and dark cyan data points respectively. b) Time dependence of the 425 nm CD response of the Bfd cluster in the presence of xanthine oxidase, hypoxanthine with either 10 µM Fe^2+^ (black), 2 mm glutathione (red), or both 10 µM Fe^2+^ and 2 mm glutathione (blue). All data were recorded in the presence of 210 µM O_2_. c) CD spectra of Bfd following the treatments in (b), grey trace shows that of the protein prior to treatment for comparison.

Loss of only 6 µM (30%) Bfd clusters in the presence of 40 µM superoxide and 57 µM peroxide demonstrated that degradation of the [2Fe–2S] is not stoichiometric under the conditions employed. However, the labile iron pool of the cytoplasmic environment is thought to be tightly regulated in part to avoid deleterious reactions with peroxide and superoxide generating the more reactive hydroxyl radical. In addition, this environment is rich in glutathione (GSH) or equivalent low molecular weight thiols thought to chelate the majority of this labile iron.^[^ ^40^ ^]^ Therefore, in order to investigate the effect of ROS in combination with intracellular iron on Bfd cluster stability, 10 µM Fe^2+^ and 2 mm GSH, typical of estimates of intracellular concentrations,^[^ ^40^, ^41^, ^42^, ^43^, ^44^ ^]^ were added individually and in combination to assays of Bfd cluster stability to assess their influence on ROS‐mediated damage. Control experiments showed that, in the absence of O_2_, the Bfd cluster was stable in the presence of 2 mm GSH and that > 95% of cluster CD intensity was retained following exposure to 10 µM Fe^2+^ for 1 h (Figure 3).

Stability was decreased in the presence of 210 µM O_2_ with both 2 mm GSH and 10 µM Fe^2+^ now inducing decay of CD intensity equating to loss of 8% (1.6 µM) of Bfd clusters with an initial velocity equating to loss of 1.3 nm cluster s^−1^ (Figure 3). In the presence of ROS generated by the xanthine oxidase system, 10 µM Fe^2+^ induced decay of the Bfd cluster at a rate of 2 nm s^−1^ but the more striking effect occurred in the presence of 2 mm GSH, with cluster degradation increased to 60% (12 µM) with an initial velocity of cluster loss of 9 nm s^−1^. The destabilising effects of Fe^2+^ and GSH appear to be cumulative with incubation of Bfd with 10 µM Fe^2+^ and 2 mm GSH in the presence of xanthine oxidase generated ROS leading to loss of 90% of clusters with an initial velocity of 20 nm s^−1^ (Figure 3). Therefore, in the presence of GSH concentrations presumed to mimic those encountered in vivo, Bfd clusters are rapidly degraded in the presence of ROS and this is exacerbated by the presence of Fe^2+^.

### Peroxide is the Main Agent of Bfd Cluster Damage

In order to determine which of the ROS generated by the xanthine oxidase system was the major contributor to Bfd cluster degradation, assays were repeated in the presence of the protective enzymes catalase, which catalyses the conversion of H_2_O_2_ to water and O_2_, and superoxide dismutase (SOD), which catalyses the conversion of superoxide to O_2_ and O_2_^2−^. Figure S2 shows the effect of 500 U mL^−1^ of catalase and 100 U mL^−1^ of SOD on the concentration of xanthine oxidase‐generated peroxide detected using the Amplex Red assay and on the concentration of superoxide detected via the reduction of cytochrome *c*. The data show that catalase reduced the concentration of peroxide detected by approximately 90%, whilst the action of SOD increased the concentration of peroxide detected by 13 µM. In contrast, the extent of cytochrome *c* reduction following exposure to xanthine oxidase and hypoxanthine in the presence of SOD indicated approximately 40% of superoxide (16 µM) was not eliminated by SOD prior to heme reduction (Figure S2D).

**Figure 4:**
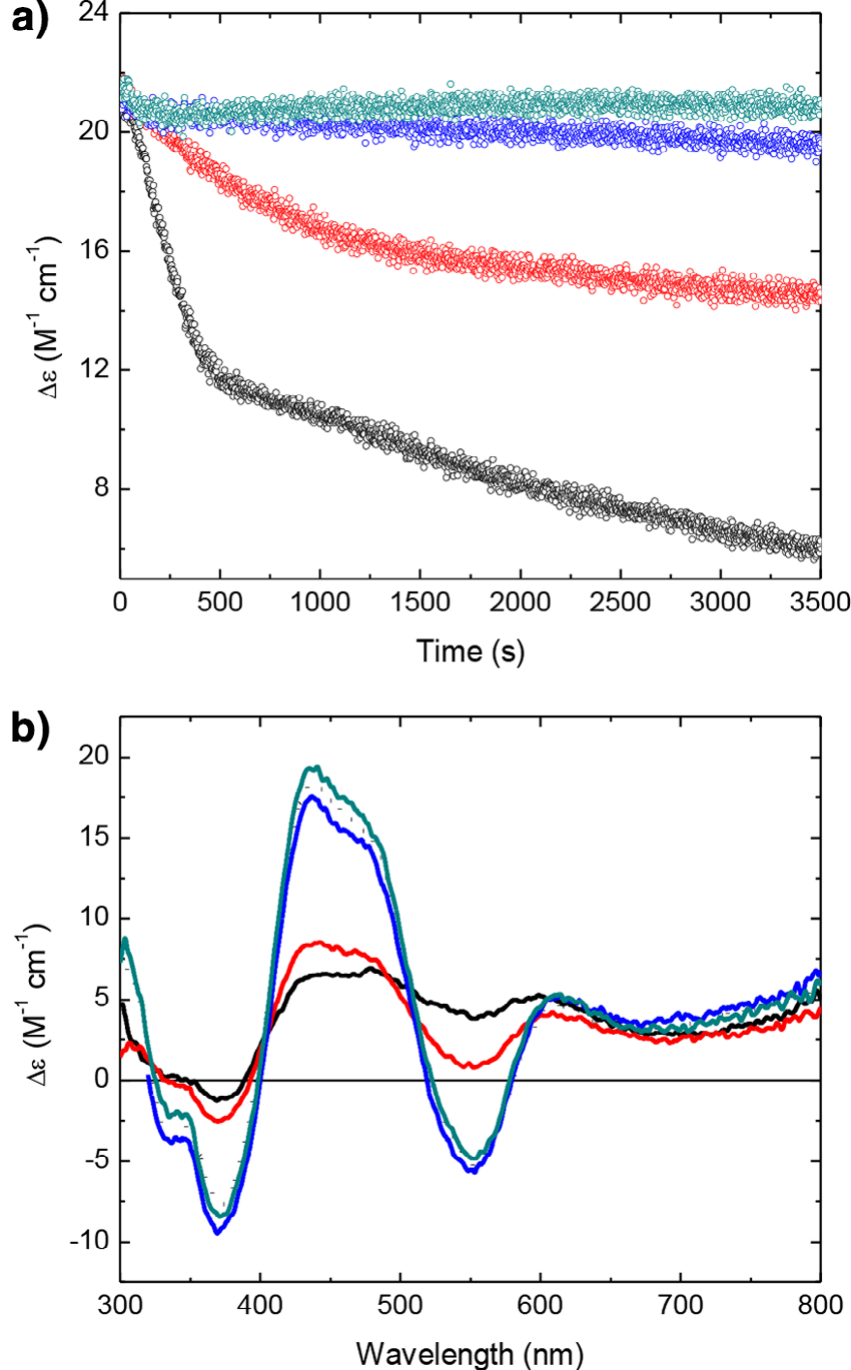
The protective effect of catalase and SOD on ROS‐mediated damage to Bfd. a) Time dependence of the 425 nm CD intensity due to the cluster of Bfd in the presence of xanthine oxidase, hypoxanthine, 10 µM Fe^2+^ and 2 mm GSH with no ROS detoxifying enzymes present (black), or in solution containing 100 U mL^−1^ SOD (red), 500 U mL^−1^ catalase (blue) or both SOD and catalase (dark teal). b) CD spectra following each of the treatments described in (a) together with that of Bfd prior to ROS exposure for comparison (dashed black trace).

The effect of the addition of catalase and SOD on Bfd cluster degradation by xanthine oxidase‐generated ROS in the presence of 10 µM Fe^2+^ and 2 mm GSH is shown in Figure 4. The data show that the Bfd cluster was stable over a period of an hour with both enzymes present. However, the Bfd cluster was equally stable when only catalase was added to the assay mixture. In the presence of SOD as the only protective enzyme, the decay of the cluster was similar to that observed with no ROS‐detoxifying enzymes, but Fe^2+^ omitted from the assay solution (compare Figures 4a and 3b). The stabilising effect of catalase on the Bfd cluster suggests that peroxide is the major causative agent of damage. Given the presence of Fe^2+^ in the assay solution, the most likely mechanism of cluster degradation is via hydroxyl radical formation due to oxidation of the metal by peroxide. Superoxide then acts as a reducing agent, regenerating Fe^2+^ more effectively than the GSH present, hence the secondary protective effect of SOD. The model of peroxide‐induced damage being caused by its reaction with Fe^2+^ to generate hydroxyl radicals is supported by the stability of the cluster towards exogenously added peroxide in the absence of Fe^2+^ in solution (Figure S4).

### ROS Exposure Impairs the Ability of Bfd to Support Reductive Iron Release from Bfr

Bfd functions to shuttle electrons from NADPH into the mineral core of Bfr, solubilising it by reduction to Fe^2+^ to facilitate release into the cytoplasm under conditions of iron limitation. This activity is underpinned by redox cycling of the Bfd cluster between the [2Fe–2S]^+^ and [2Fe–2S]^2+^ oxidation states.^[^ ^32^, ^33^ ^]^ ROS‐induced damage to the cluster is therefore predicted to eliminate the ability of Bfd to mediate iron release from Bfr. However, in vitro demonstration of this impairment of function is not straightforward. Use of the xanthine oxidase system to produce ROS imposes the need for aerobic conditions for enzyme turnover that are incompatible with the use of reduced Bfd to mediate iron release from Bfr, as the cluster is readily oxidised by O_2_. Since reduced cluster is required to support Bfr core reduction, termination of iron release due to the presence of xanthine oxidase‐generated ROS would be indistinguishable from that due to oxidation of the Bfd cluster. Consequently, because in situ generation of ROS during iron release assays was impractical, samples of Bfd were pre‐exposed to xanthine oxidase‐generated ROS then incubated with dithionite under anaerobic conditions and the effect of this treatment on the ability of Bfd to support iron release from Bfr was compared to equivalent treatment of FMN.

**Figure 5:**
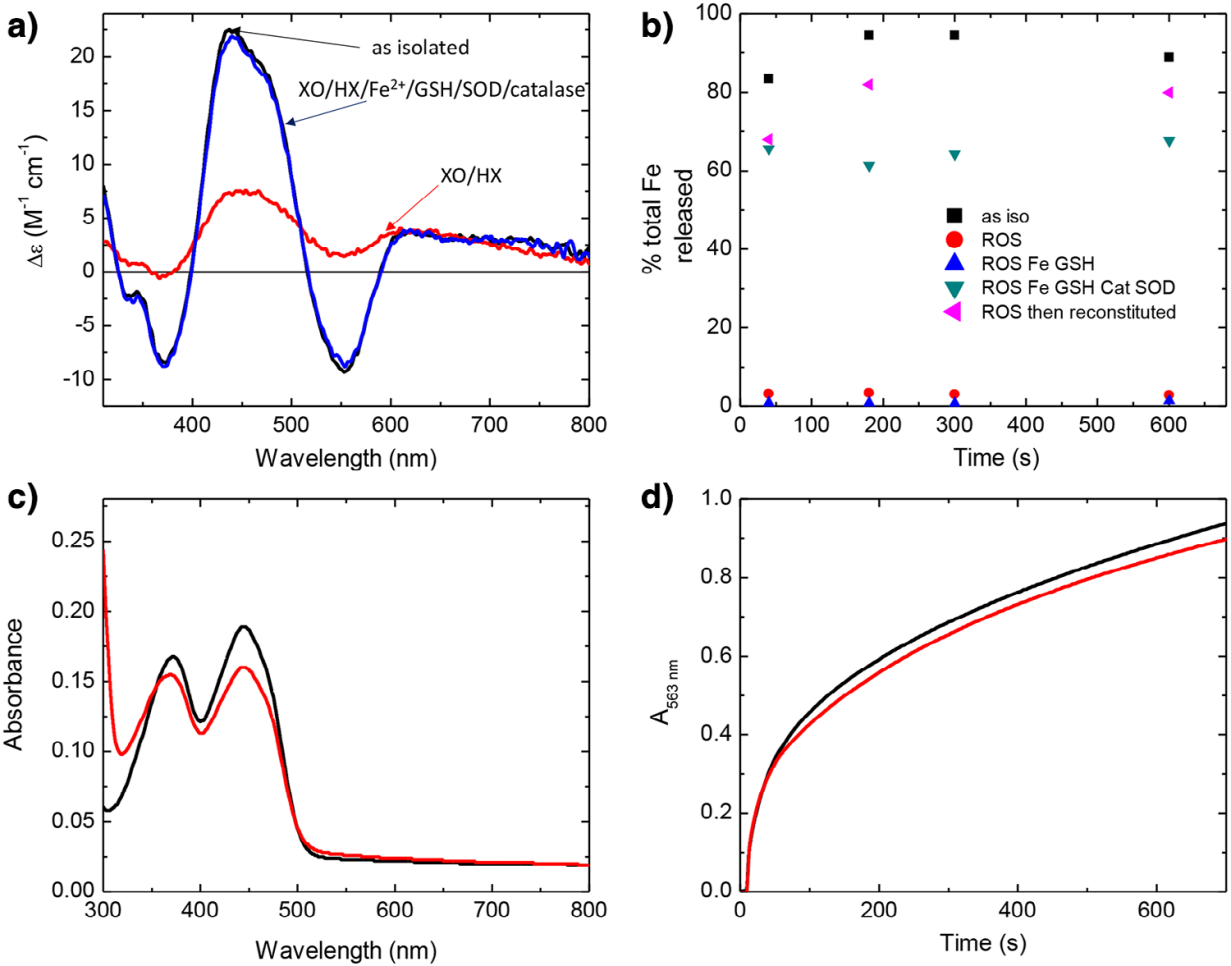
The impact of ROS exposure on the capacity of Bfd and FMN to support reductive iron release from Bfr. a) The CD spectrum of as‐isolated 60 µM Bfd (black), following exposure to xanthine oxidase‐generated ROS for 1 h (red), and following incubation with 1 mm hypoxanthine, xanthine oxidase, 10 µM Fe^2+^, 2 mm glutathione, 500 U mL^−1^ catalase, and 100 U mL^−1^ SOD for 1 hour (blue). b) Plots of instantaneous increases in 563 nm absorbance as a function of time on addition of 1 mm ferrozine to solutions containing 50 nm Bfr loaded with 1200 equiv. of Fe^3+^ per protein cage and: 60 µM reduced as‐isolated Bfd (black); 60 µM reduced Bfd pre‐exposed to xanthine oxidase‐generated ROS (red); 60 µM reduced Bfd pre‐exposed to xanthine oxidase‐generated ROS in the presence of 10 µM Fe^2+^ and 2 mm glutathione (dark cyan); 60 µM reduced Bfd pre‐exposed to xanthine oxidase‐generated ROS in the presence of 10 µM Fe^2+,^ 2 mm glutathione, 500 U mL^−1^ catalase and 100 U mL^−1^ SOD (blue) or 60 µM reduced Bfd pre‐exposed to xanthine oxidase‐generated ROS prior to reconstituting the [2Fe–2S] cluster (magenta). Time zero is the point at which Bfd was added. c) Absorbance spectra of freshly prepared FMN (black) and FMN exposed to xanthine oxidase‐generated ROS for 1 h (red). d) Increase in 563 nm absorbance of solutions containing 1 mm ferrozine and 50 nm Bfr loaded with 1200 equiv. of Fe^3+^ per protein cage as a function of time following the addition of 40 µM reduced FMN (black) or reduced FMN pre‐exposed to xanthine oxidase‐generated ROS for 1 h (red).

Assays of iron release from Bfr required a higher concentration of Bfd (60 µM) than studies of cluster stability. Since ROS‐mediated damage was O_2_‐limited, even at the lower Bfd concentration, samples for iron‐release assays were exposed to xanthine oxidase‐generated ROS whilst stirring in air for 60 min to continuously introduce dissolved O_2_. Figure 5 shows the effect of this treatment on the optical properties of 100 µM solutions of Bfd and FMN. Exposure of Bfd to ROS generated in this fashion in the presence of 2 mm GSH and 10 µM Fe^2+^ led to almost complete (∼85%) loss of cluster (as shown in Figure 4b), as judged by loss of CD intensity. In contrast, equivalent treatment in the presence of 500 U mL^−1^ catalase and 100 U mL^−1^ SOD had a negligible effect on cluster CD intensity (Figure 5a). The ROS‐exposed samples were subsequently incubated with 100 µM sodium dithionite, to reduce remaining intact Fe–S cluster, then exchanged by centrifugation into a mixed buffer system (see Materials and Methods) to remove GSH, iron and unreacted dithionite prior to use in iron‐release assays.

To allow direct comparison with FMN, a further sample of Bfd was exposed to xanthine oxidase‐generated ROS but in the absence of Fe^2+^ and GSH (iron and GSH cannot be separated from FMN by centrifugation meaning the added Fe^2+^ would be chelated by ferrozine in addition to that released from Bfr, whilst the GSH present could potentially act as both reducing agent and iron chelator), again resulting in almost complete loss of the cluster (Figure 5a). FMN has no appreciable CD intensity at the concentrations used here, but its absorbance spectrum was not significantly altered by exposure to xanthine oxidase‐generated ROS. The changes observed were consistent with reduction of a small fraction of the sample by superoxide (Figure 5c).

The effect of the above treatments on the rate and extent of iron release from Bfr mediated by Bfd and FMN are also shown in Figure 5. Chelation of Fe^2+^ liberated from the Bfr core by ferrozine leads to the formation of a coloured complex with *ε*_563 nm_ of 27900 m ^−1^ cm^−1^. The increase in absorbance at this wavelength can therefore be converted to a percentage of total Bfr‐encapsulated iron released as a function of time. As expected, from the degree of cluster degradation, Bfd exposed to ROS with or without Fe^2+^ and GSH present was unable to support reduction of the Bfr mineral core (Figure 5b). However, ROS exposure even in the presence of catalase and SOD resulted in impaired activity in iron‐release assays, despite the spectroscopic signature of the ferredoxin cluster being unaffected. In this sample, iron release plateaued at approximately 70% of the total internalised content of Bfr compared to release of all mineralised iron when using a control sample of reduced Bfd that had not been pre‐exposed to ROS. In both instances release of iron was essentially complete in under 60s. The relatively small decrease in activity observed for Bfd pre‐exposed to ROS in the presence of SOD and catalase is likely to be due either to re‐oxidation of released Fe^2+^ at Bfr FoCs or the [2Fe–2S]^+^ cluster itself driven by small quantities of residual H_2_O_2_,^[^ ^45^ ^]^ or damage to Bfd that occurs during ROS exposure that inhibits electron transfer activity without significantly altering the optical properties of the [2Fe–2S] cluster. Importantly, Bfd inactivated by ROS‐induced degradation of the [2Fe–2S] cluster had almost full activity restored by reconstitution of the cluster^[^ ^46^ ^]^ (Figures S5, 5), demonstrating that the majority of loss in activity was due to cluster degradation rather than non‐specific oxidative damage to the protein. Iron release driven by reduced FMN was much slower, being incomplete even after 10 min (Figure 5d). In contrast to Bfd, neither the rate nor the extent was significantly affected by pre‐exposure of the nucleotide to ROS.

## Conclusions and Perspective

Bfd is absolutely required in order to mobilise the iron contained within the mineral core of Bfr.^[^ ^37^ ^]^ Contrary to initial reports,^[^ ^47^ ^]^ it has now been shown that it is the holo form of the Bfd that supports iron release, via the redox cycling of its [2Fe–2S] cluster. This places the requirement on cells that are deficient in iron to synthesise an iron‐containing co‐factor in order to access their Bfr‐encapsulated stores of this metal. We postulate that bacteria may have evolved to be reliant on the presence of Fe–S clusters for mobilisation of stored iron from Bfr due to the susceptibility of these co‐factors to oxidative damage. Inactivation of the cluster by ROS‐mediated damage, most likely via oxidation of sulfide and/or cysteine thiols, would shut down release of iron into the cytoplasm, which would otherwise exacerbate oxidative stress. However, the activity of catalase and SOD protect Bfd from ROS‐mediated damage, preserving its ability to support release of iron from Bfr. Therefore, access to Bfr iron stores would only be severely impacted if ROS accumulate to concentrations that cannot be adequately detoxified by catalase and/or SOD. Furthermore, we demonstrate that the capacity of Bfd to support reductive iron release from Bfr is more susceptible to impairment by ROS than that of FMN, an iron‐free redox active co‐factor that is also capable of reducing Bfr cores in vitro.

Bfr proteins exhibit a balance between iron storage and iron release activities, with one usually dominant over the other, depending on the cellular iron status. Under oxidative stress that overwhelms the catalase/SOD detoxification response, iron release capability would be lost along with the Bfd cluster. Bfr would retain its iron mineralisation activity, and so excess Fe^2+^ resulting from, e.g., damage to Fe–S cluster proteins, would be a substrate for mineralisation (in competition with other iron‐requiring processes, such as SUF Fe–S cluster synthesis^[^ ^48^ ^]^). We note that the very rapid reaction of Fe^2+^ at the Bfr ferroxidase centre with peroxide^[^ ^45^ ^]^ may indeed contribute to cellular detoxification of ROS under such oxidative stress conditions.

Bacterial genomes often encode more than one ferritin, and Bfrs often do not constitute the major metabolic iron store in prokaryotes.^[^ ^49^ ^]^ However, the high degree of co‐occurrence of Bfr and Bfd in bacteria, together with the high level of sequence identity of key residues at the Bfr:Bfd interface across many examples of these proteins (Figure 1c) suggests that the Bfd‐dependent mechanism for iron release is widespread.

Structure‐function relationships in Fe–S cluster‐containing proteins related to oxidative stress are known to be widespread. For example, the [4Fe–4S] cluster‐binding protein IRP1 (cytoplasmic aconitase) regulates iron metabolism. Low iron and/or oxidative stress results in degradation of the cluster (by ROS or RNS, generated downstream of ROS), exposing an RNA‐binding site and enabling the protein to bind to the iron responsive element in the mRNA of transferrin receptor and ferritin thereby modulating their translation.^[^ ^50^ ^]^ Oxidation or nitrosylation of the [2Fe–2S] cluster of SoxR activates the protein to trigger SoxS production,^[^ ^51^ ^]^ which in turn upregulates transcription of genes encoding oxidative defence mechanisms such as MnSOD and DNA repair. Here, we propose a different phenomenon of regulation at the protein level via susceptibility of a [2Fe–2S] co‐factor, which is essential for function, to oxidative damage. This loss of function is more akin to that described following the binding of RNS to enzymes such as cytochrome P450^[^ ^52^ ^]^ and nitric oxide synthase.^[^ ^53^ ^]^

The coordinated transcriptional regulation of iron and O_2_ metabolism in aerobic organisms is well established. For example, in *E. coli*, levels of the global iron regulator Fur are regulated in response to oxidative stress. Transcription of *fur* is activated by OxyR when it is oxidised by H_2_O_2_, and also by SoxRS in response to superoxide inducers.^[^ ^54^, ^55^ ^]^ The effect of this is to repress iron‐uptake systems and iron release (down‐regulation of *bfd* expression), thus limiting cellular iron levels under conditions where ROS levels are elevated.^[^ ^56^ ^]^ It was shown that, in an *E. coli* mutant lacking peroxidases and catalase activity, accumulating H_2_O_2_ resulted in complete de‐repression of the Fur regulon, suggesting that the Fur‐Fe^2+^ complex was damaged under ROS stress.^[^ ^57^ ^]^ We postulate that this potential vulnerability, where ROS‐induced damage to the Fur complex decouples the transcriptional iron homeostasis response, is in part tempered by the direct protein regulation via Bfd where loss of function due to destruction of the redox active co‐factor represents an additional, and more immediate, response compared to the genetic regulatory responses that down‐regulate Bfd under oxidative stress conditions.^[^ ^58^ ^]^

The intermolecular interactions of Fe–S cluster‐containing proteins are also known to be influenced by cluster integrity. For example, in the base excision repair enzyme endonuclease III, binding of the [4Fe–4S] cluster positions conserved basic residues within the cluster‐binding loop for favourable interaction with the phosphate backbone of DNA.^[^ ^59^ ^]^ Similarly, binding to cognate operator DNA sequences of several Fe–S cluster‐containing transcriptional regulators, such as FNR,^[^ ^60^ ^]^ NsrR,^[^ ^61^ ^]^ and RirA,^[^ ^62^ ^]^ are all cluster‐dependent. Comparison of the structure of *P. aeruginosa* Bfd to that of its complex with Bfr^[^ ^32^, ^63^ ^]^ reveals that very little re‐organisation of the Bfd structure occurs on complex formation. However, the cluster‐binding cysteine residues are located on loops L1 and L3 that comprise the interaction surface with Bfr (Figure 1b), meaning that cluster binding may well lock these loops in position for favourable interaction with the outer surface of Bfr. It is therefore reasonable to assume that the consequences of cluster degradation are two‐fold, with damage lowering the binding affinity of Bfd for Bfr in addition to eliminating redox activity in the former.

Exploiting the vulnerability of Fe–S clusters to facilitate regulation at the protein level may extend beyond the Bfd:Bfr interaction. Some ferric iron reductases, enzymes dedicated to the reductive release of iron sequestered using siderophores, employ a ferredoxin to reduce their flavin co‐factors. Others with specificity for hydroxamate‐based siderophores, such as *E. coli* FhuF, directly employ [2Fe–2S] clusters as their redox‐active co‐factor.^[^ ^64^, ^65^ ^]^ Whilst flavin co‐factors that accept electrons from NAD(P)H are the most widespread class of ferric iron reductase, the existence of examples reliant on Fe–S clusters for activity raises the intriguing possibility that termination of activity due to ROS damage of these co‐factors may extend to some routes of iron acquisition, in addition to the accessing of stored iron. We propose that bacteria employ such a strategy as a failsafe to evade increased levels of ROS and RNS under oxidative stress, such as that produced by host immune responses to pathogen invasion.^[^ ^66^, ^67^ ^]^

A further intriguing possibility is that the host organisms also exploit this same strategy to prevent an excessive immune response,^[^ ^68^ ^]^ i.e., that Fe–S cluster proteins involved in the immune response function as “biological fuses”. It was also proposed that such a biological fuse function of Fe–S proteins/enzymes is important in feedforward and feedback mechanisms that underpin the ability of the innate immune response to restrict viral replication.^[^ ^68^ ^]^

The susceptibility of Fe–S co‐factors to damage resulting from oxidative (and nitrosative) stress raises the question of why they were in large part retained in nature following the oxygenation of the atmosphere. We propose that, in part, this was to exploit their sensitivity to oxidative stress in order to shut down potentially lethal feedback loops. We anticipate the discovery of further examples of such Fe–S cluster fuses, along with alternative means by which aerobic organisms guard against increasing cellular concentrations of Fe^2+^ under conditions of oxidative stress.

## Author Contributions

J.M.B.: conceptualization, investigation, formal analysis, writing — original draft. A.M.C.: investigation, formal analysis; Z.B.: investigation, formal analysis. S.C.A.: investigation, writing — review and editing. N.L.B.: conceptualization, funding acquisition, supervision, writing — review, and editing.

## Conflict of Interests

The authors declare no conflict of interest.

## Supporting information



Supporting Information

## Data Availability

The data that support the findings of this study are available from the corresponding author upon reasonable request.
