# Supplementary material for: The Iron–Sulfur Cluster of Bacterioferritin‐Associated Ferredoxin (Bfd): a “Biological Fuse” that Prevents Oxidative Damage to Cells?
Source: Angew Chem Int Ed Engl. 2025 Jun 25;64(34):e202511340. doi: 10.1002/anie.202511340 (PMC12363616; doi:10.1002/anie.202511340)
Supplement: Supplementary file 1 — Supporting Information [file ANIE-64-e202511340-s001.pdf]

Supporting Information

The Iron-Sulfur Cluster of Bacterioferritin-Associated Ferredoxin (Bfd): A ‘Biological Fuse’ that Prevents Oxidative Damage to Cells?

Justin M. Bradley, Aiden M. Carter, Zinnia Bugg, Simon C. Andrews and Nick E. Le Brun

Materials and Methods
All procedures were performed in 20 mM phosphate buffer, pH 7.4, unless otherwise stated.

Protein production. Bfd and heme-loaded Bfr containing a mineral core of 1200 equivalents of Fe^{3+} were produced as described elsewhere[1]. Xanthine oxidase, horse heart cytochrome *c*, catalase and superoxide dismutase were purchased from Merck.

Spectroscopic measurements. Absorbance spectra and associated measurements of absorbance as a function of time were recorded on a Hitachi U2900 spectrophotometer. Circular dichroism (CD) spectra and associated measurements of CD as a function of time were recorded on a Jasco J810 circular dichrograph. Equivalent magnetic circular dichroism measurements were also recorded on the Jasco J810 circular dichrograph, but with the sample chamber equipped with a Jasco PMCD-586 permanent magnet generating a field of 1.6 Tesla at the sample.

Impact of ROS on Bfd cluster stability. For cluster-stability assays, ROS were generated *in situ* via the activity of xanthine oxidase. 22 mU/mL of xanthine oxidase was added to solutions of 20 μM Bfd that also contained 500 U/mL catalase, 100 U/mL superoxide dismutase, 1 mM glutathione and 10 μM Fe^{2+}, as required. Xanthine oxidase reactivity was initiated by the addition of hypoxanthine to a final concentration of 1 mM. Cluster stability under the conditions surveyed was deduced from the rate and extent of the decrease in 336 nm absorbance and/or CD at 437 nm. The concentration of ROS generated by the xanthine oxidase/hypoxanthine system was quantified as follows. Hypoxanthine was added to a final concentration of 1 mM to solutions of 100 μM horse heart cytochrome *c*, 22 mU/mL xanthine oxidase and 500 U/mL catalase and the concentration of superoxide generated determined by the extent of reduction of the ferric cytochrome *c* hemes. Peroxide generation was quantified by adding hypoxanthine to a final concentration of 1 mM to 22 mU/mL xanthine oxidase in the presence of the Amplex Red (Thermo Fisher) kit components, and the extent of oxidation of the dye compared to a calibration plot constructed using serial dilution of a standard peroxide solution.

Assays of Fe^{2+} release from Bfr. Iron-release assays were conducted under anaerobic conditions in a mixed buffer system of 10 mM potassium acetate, 10 mM MES, 10 mM MOPS, 10 mM Tris, 200 mM NaCl, pH 6.0. Bfr loaded with 1200 equivalents of Fe^{3+} was diluted to a concentration of 0.05 μM such that the concentration of iron in the assay solution was 60 μM. FMN and Bfd were reduced by anaerobic incubation with sodium dithionite. Dithionite was titrated into 100 μM solutions of the former until approximately 90% of the flavin was reduced, as judged by bleaching of the absorbance feature at 446 nm (ε = 12,200 M^{-1} cm^{-1}). FMN solutions contained catalase at 500 U mL^{-1} to remove any residual peroxide from xanthine oxidase activity as peroxide is an extremely effective oxidant of Bfr ferroxidase centres and its presence would therefore interfere with assays of reductive release of iron from the protein.

FMN-mediated reductive release of mineralised iron was initiated by injection of Bfr into an assay solution containing 55 μM reduced FMN and 1 mM 3-(2-Pyridyl)-5,6-diphenyl-1,2,4-triazine-p,p'-disulfonic acid (ferrozine). Rate and extent of iron release was determined by monitoring the increase in 563 nm absorbance as a function of time following addition of the ferritin. For Bfd-mediated reductive release of iron,100 μM solutions of Bfd were equilibrated with

100 μM sodium dithionite, followed by exchange into dithionite-free buffer by centrifugation over a 3 kDa molecular weight cut off cellulose membrane (Millipore). Iron release was initiated by mixing Bfr and Bfd to final concentrations of 0.055 and 66 μM, respectively, in 360 μL aliquots of assay solution. Rate and extent of iron release was determined by monitoring the instantaneous increase in 563 nm absorbance following injection of 40 μL of a 10 mM ferrozine solution at defined timepoints after initiating iron release.

Reconstitution of the Bfd [2Fe-2S] cluster. A [2Fe-2S] cluster was reconstituted on apo Bfd by incubating the protein with Fe^{2+} and dithiothreitol (DTT) as reducing agent with the required sulfide generated by the action of the cysteine desulferase NifS on L-cysteine. 200 μM apo Bfd was generated by exposing the as isolated protein to ROS generated by xanthine oxidase as described above then separating the protein from reagents and cluster breakdown products by passage down a PD-10 de-salting column (Cytiva) equilibrated with 20 mM phosphate buffer pH 7.4. Apo Bfd was then incubated with 2.5 mM DTT, 2.8 mM Fe^{2+}, 1 mM L-cysteine and 500 nM NifS at 30 ˚C for 150 min and reconstitution of the [2Fe-2S] cluster monitored by absorbance (Fig. S5). The reconstitution reaction was terminated by separating Bfd from the Fe^{2+}, DTT and L-cysteine using a PD-10 column once the absorbance spectrum began to show evidence of light scattering due to formation of FeS.

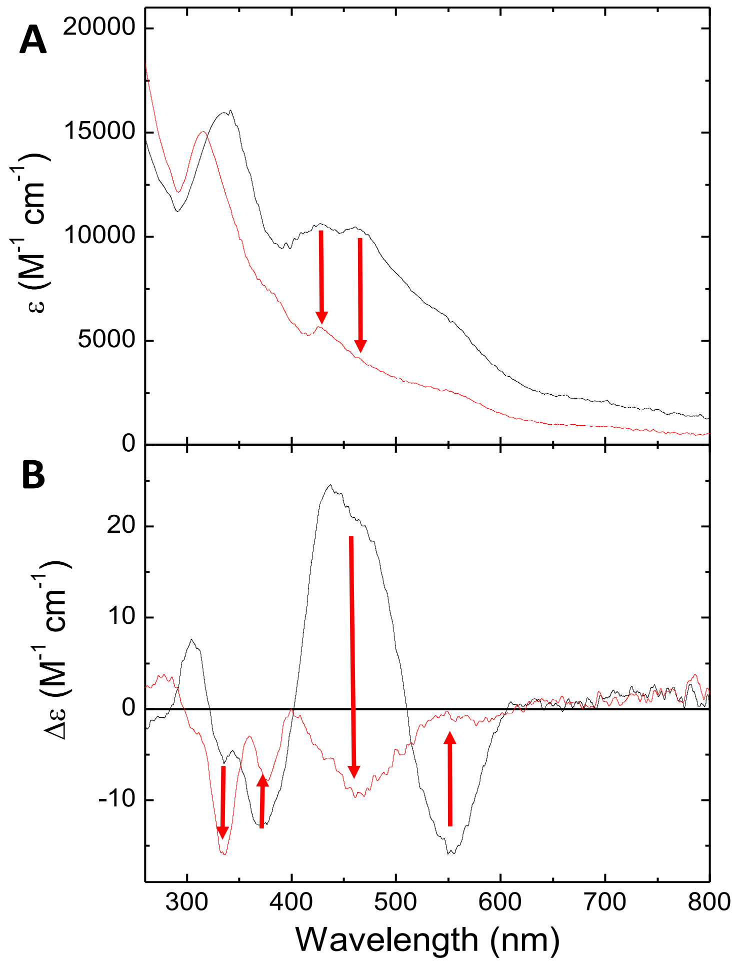

Figure S1. Changes in the optical spectra of *E. coli* Bfd on reduction of the [2Fe-2S] cluster. (**A**) UV-visible and (**B**) circular dichroism spectra of Bfd with the [2Fe-2S] cofactor is the +2 (black traces) and +1 (red traces) oxidation state. Red arrows indicate the change in intensity upon reduction of the cluster.

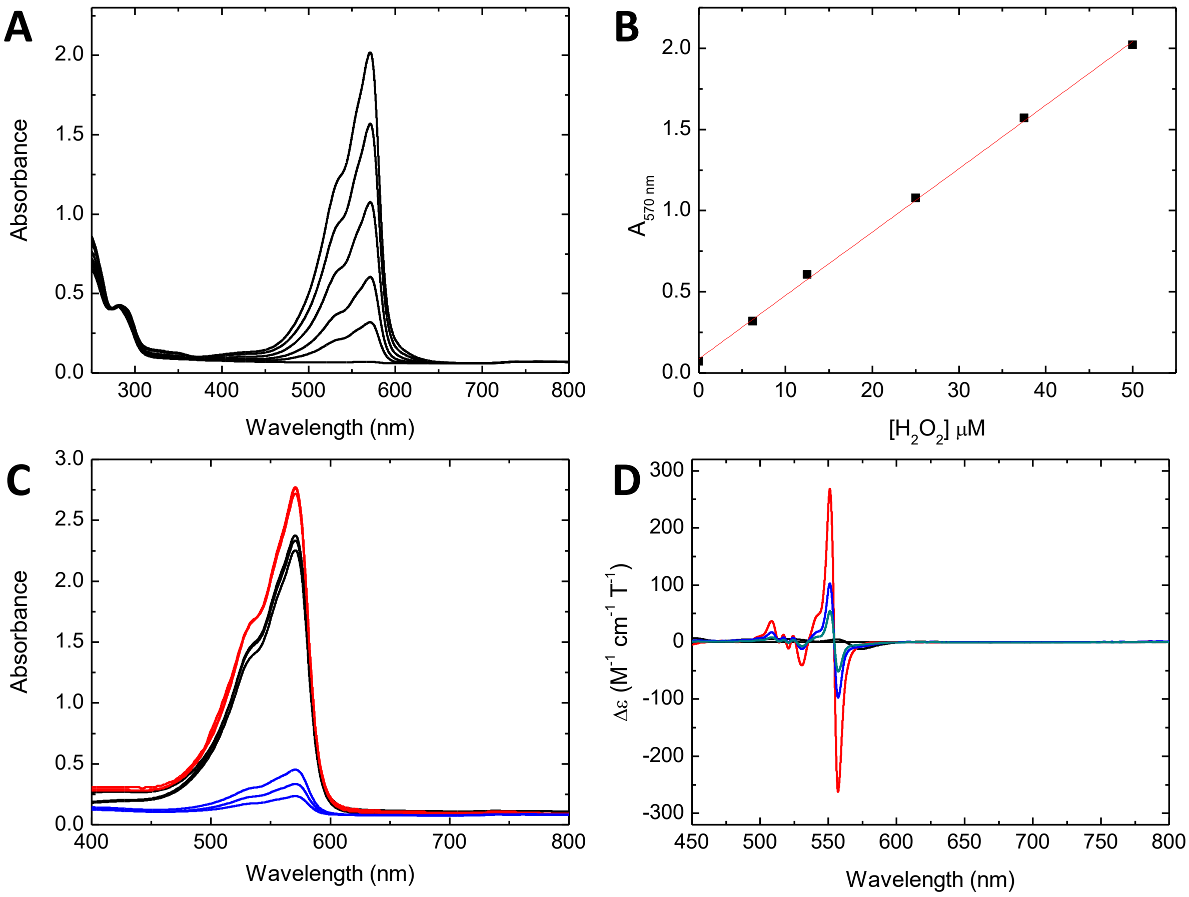

Figure S2. Quantification of ROS generated by xanthine oxidase. (**A**) Absorbance spectra of assay solutions following incubation of Amplex Red with standard solutions containing 0, 6.25, 12.5, 25, 37.5 and 50 μM H_2O_2 in the presence of horse radish peroxidase, showing the increase in 570 nm absorbance due to oxidation of Amplex Red dye to resorufin. (**B**) Calibration plot showing the linear relationship between the intensity of 570 nm absorbance and concentration of H_2O_2 in the assay solutions from (A). (**C**) Absorbance spectra of Amplex Red assay solutions containing 22 mU/mL xanthine oxidase, 1 mM hypoxanthine (black), together with 100 U/mL superoxide dismutase (red) or 500 U/mL catalase (blue), measurements performed in triplicate. (**D**) Magnetic circular dichroism spectra of fully oxidised (black) and fully reduced (red) 100 μM cytochrome *c* together with those following incubation of the fully oxidised protein with 22 mU/mL xanthine oxidase, 1 mM hypoxanthine, 500 U/mL catalase with (teal) or without (blue) 100 U/mL superoxide dismutase present. Spectra demonstrate the extent of reduction of the cytochrome *c* hemes due to superoxide generated by the xanthine oxidase system.

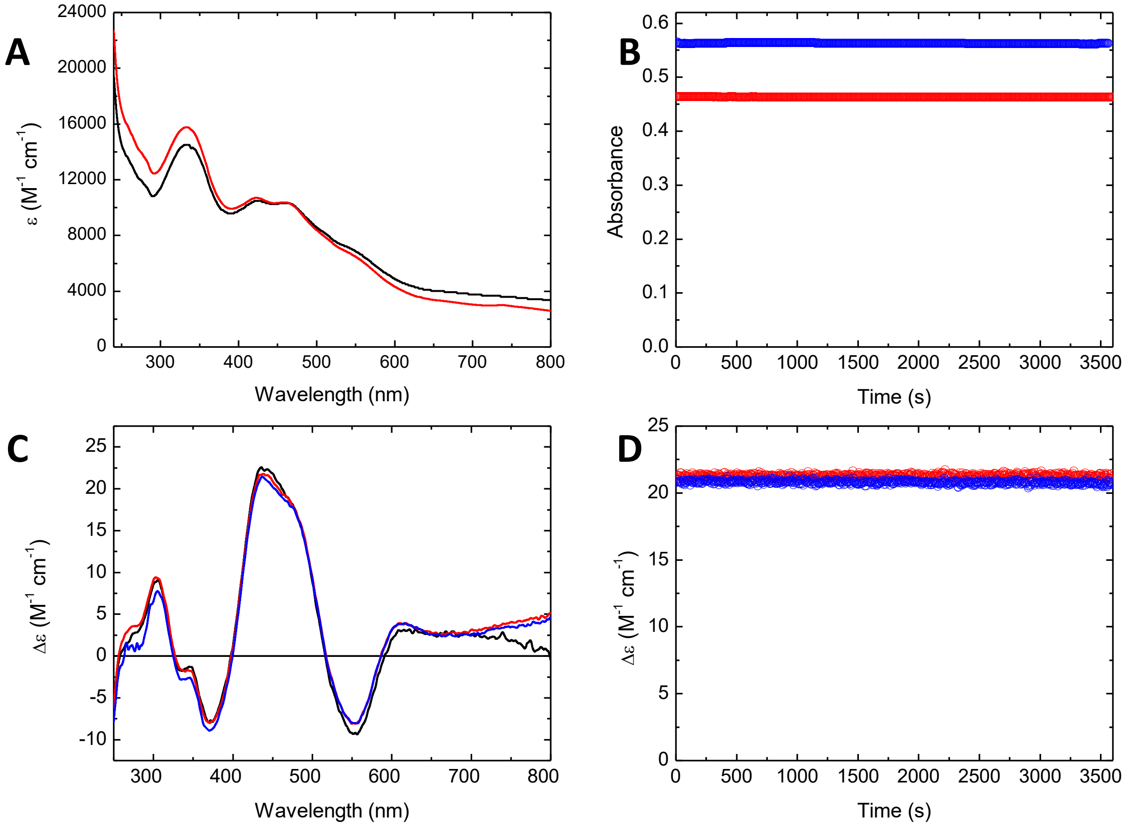

Figure S3. Stability of the Bfd cluster to O_2 and the products of xanthine oxidase activity. (**A**) Absorbance spectra of anaerobic Bfd (black) and following exposure to 125 µM O_2 for one hour (red). (**B**) Absorbance at 335 nm as a function of time following exposure of Bfd to 125 µM O_2 (red) or 125 µM O_2, 1 mM xanthine, 1 mM uric acid (blue). (**C**) CD spectra of anaerobic Bfd (black) and following exposure to 125 µM O_2 for one hour (red) or 125 µM O_2, 1 mM xanthine, 1 mM uric acid for 1 hour (blue). (**D**) CD intensity at 435 nm as a function of time following exposure of Bfd to to 125 µM O_2 (red) or 125 µM O_2, 1 mM xanthine, 1 mM uric acid (blue).

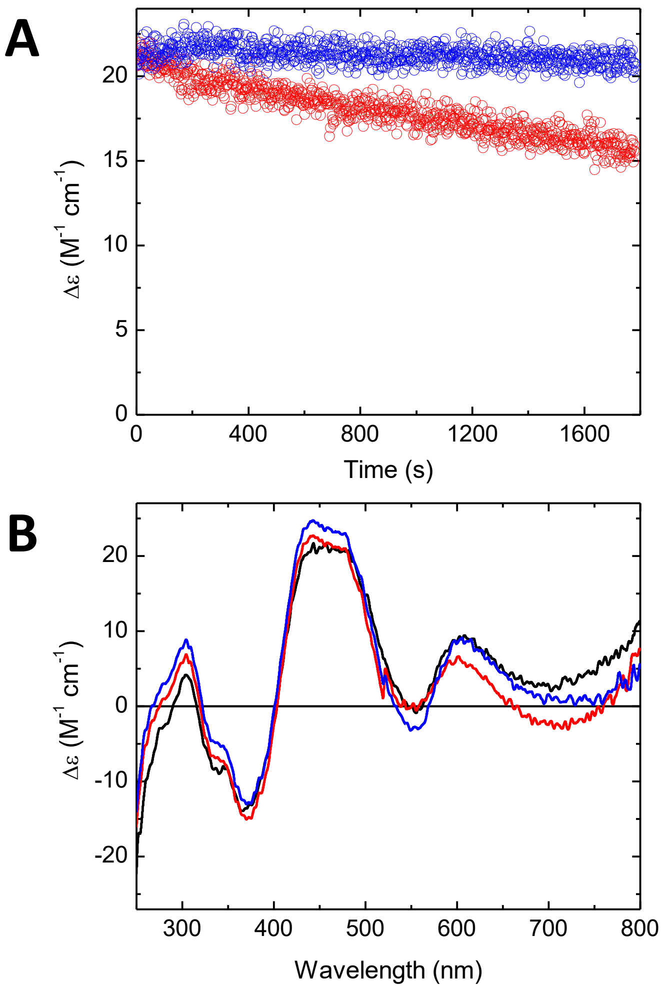

Figure S4. Stability of the Bfd cluster in the presence of peroxide. (**A**) 435 nm CD intensity of the Bfd [2F-2S]-cluster as a function of time following the addition of 250 μM (red) or 50 μM (blue) H_2O_2. (**B**) CD spectra of as-isolated Bfd (black), and Bfd following 30 min exposure to 250 μM (red) or 50 μM (blue) H_2O_2 demonstrating that variation in the 435 nm intensity over time in (**A**) is mostly associated with drift in the baseline of the instrument.

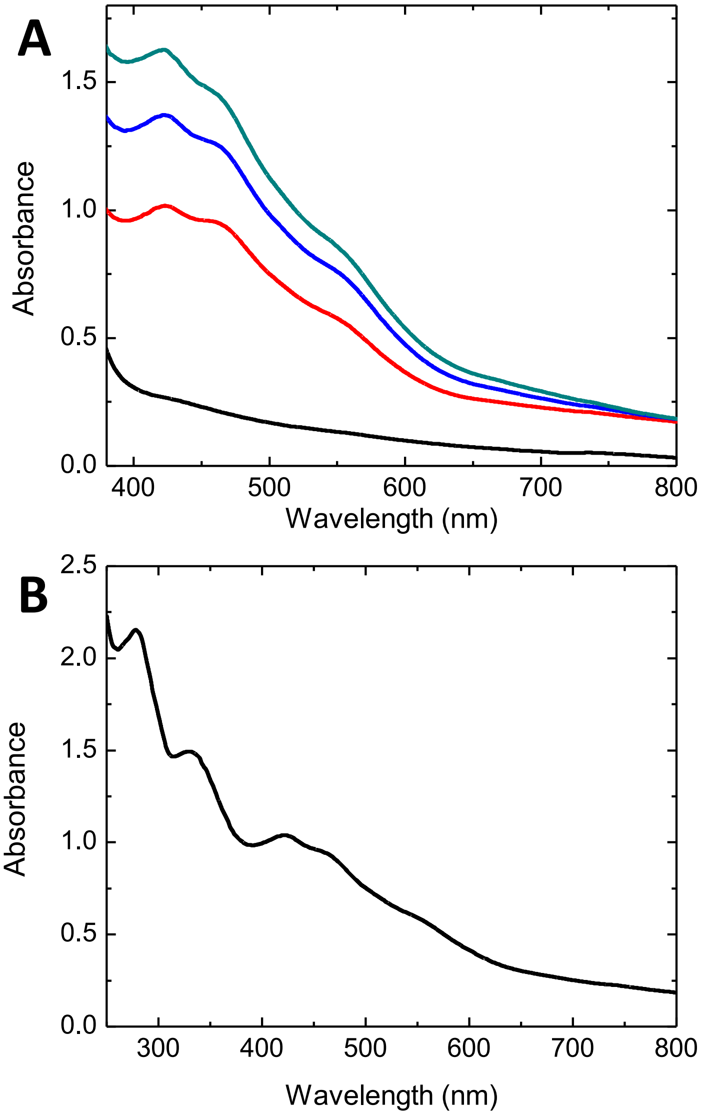

Figure S5. Reconstitution of the Bfd [2Fe-2S] cluster. (A) Absorbance spectra of apo Bfd following cluster degradation by xanthine oxidase generated ROS (black) and of Bfd with cluster bound following reconstitution involving 60 (red), 90 (blue) and 150 (cyan) min incubation with 2.8 mM Fe^{2+}, 1 mM L-cysteine, 2.5 mM DTT and 500 nM NifS at 30 ˚C. (**B**) Absorbance spectrum of Bfd incubated under the conditions in (A) for 150 min flowed by removal of small molecules by passage down a PD-10 column.

References

[1] a) J. M. Bradley, Z. Bugg, A. Sackey, S. C. Andrews, M. T. Wilson, D. A. Svistunenko, G. R. Moore, N. E. Le Brun, *Angew. Chem.Intl. Ed.* **2024**, *63*; b) J. M. Bradley, E. Gray, J. Richardson, G. R. Moore, N. E. Le Brun, *Nanoscale* **2022**, *14*, 12322-12331.
